# Fear Extinction-Based Inter-Individual and Sex Differences in Pain-Related Vocalizations and Anxiety-like Behaviors but Not Nocifensive Reflexes

**DOI:** 10.3390/brainsci11101339

**Published:** 2021-10-11

**Authors:** Peyton Presto, Guangchen Ji, Riley Junell, Zach Griffin, Volker Neugebauer

**Affiliations:** 1 Department of Pharmacology and Neuroscience, School of Medicine, Texas Tech University Health Sciences Center, 3601 4th St, Lubbock, TX 79430-6592, USA; peyton.presto@ttuhsc.edu (P.P.); guangchen.ji@ttuhsc.edu (G.J.); Riley.Junell@ttuhsc.edu (R.J.); Zach.Griffin@ttuhsc.edu (Z.G.); 2 Center of Excellence for Translational Neuroscience and Therapeutics, Texas Tech University Health Sciences Center, Lubbock, TX 79430-6592, USA; 3 Garrison Institute on Aging, Texas Tech University Health Sciences Center, Lubbock, TX 79430-6592, USA

**Keywords:** vocalizations, fear extinction, pain, sex differences

## Abstract

Inter-individual and sex differences in pain responses are recognized but their mechanisms are not well understood. This study was intended to provide the behavioral framework for analyses of pain mechanisms using fear extinction learning as a predictor of phenotypic and sex differences in sensory (mechanical withdrawal thresholds) and emotional-affective aspects (open field tests for anxiety-like behaviors and audible and ultrasonic components of vocalizations) of acute and chronic pain. In acute arthritis and chronic neuropathic pain models, greater increases in vocalizations were found in females than males and in females with poor fear extinction abilities than females with strong fear extinction, particularly in the neuropathic pain model. Female rats showed higher anxiety-like behavior than males under baseline conditions but no inter-individual or sex differences were seen in the pain models. No inter-individual and sex differences in mechanosensitivity were observed. The data suggest that vocalizations are uniquely suited to detect inter-individual and sex differences in pain models, particularly in chronic neuropathic pain, whereas no such differences were found for mechanosensitivity, and baseline differences in anxiety-like behaviors disappeared in the pain models.

## 1. Introduction

Inter-individual and sex differences have been well documented with regard to anxiety- and depression-like conditions [1,2,3] and in pain [4,5,6]. However, neural mechanisms and biomarkers related to pain vulnerability and resilience, including potential sexual dimorphisms, have yet to be fully elucidated. Intricate interactions of sensory, cognitive, and emotional-affective dimensions form the highly complex and intense experience of pain. The strong negative affective component of pain presents a challenge for effective therapeutic strategies, as patients suffering from chronic pain are at increased risk of developing mood and anxiety disorders, and vice versa [7,8,9,10]. This suggests that pain may share neurobiological mechanisms, including emotional network neuroplasticity, with negative emotions such as fear [11,12]. Fear learning and extinction networks have been implicated in neuropsychiatric disorders such as anxiety disorders, post-traumatic stress disorder (PTSD), and obsessive compulsive disorder (OCD) [13,14,15]. Vulnerability to these disorders has been predicted using fear extinction (FE) learning ability as a biomarker for inter-individual differences in the preclinical [16] and clinical [17] setting.

Behavioral studies are a crucial tool for the validation of pain mechanisms and for the assessment of potential pharmacological therapies. A variety of behavioral methods have been developed in preclinical pain models for the evaluation of traits pertaining to sensorimotor function, anxiety- and depressive-like behavior, social interactions, cognitive function, and emotional-affective responses [18]. Higher integrated pain behavior at supraspinal levels has been assessed using vocalizations. Vocalizations are an important method of communication among rodents [19], with frequencies in the audible and ultrasonic ranges. Audible vocalizations of rats in response to a noxious stimulus indicate a nociceptive reaction, whereas ultrasonic vocalizations of the 22 kHz type represent negative emotional-affective responses [20,21]. Ultrasonic vocalizations are considered an effective indicator for measuring negative emotional status and have been used in different experimental models of pain, including arthritis pain [21,22,23,24], chronic cancer pain [25,26], and neuropathic pain [27,28,29,30]. However, some have called into question the reliability of vocalizations in assessing pain-related behavior [31] and others have found that vocalizations may occur as a response to handling [32]. While a valuable behavioral measure, vocalizations as a pain assessment may be most informative when used in combination with other pain indicators [33]. Inter-individual and sex differences in audible and ultrasonic vocalizations, particularly in the context of pain and fear interactions, have not been determined.

The purpose of this study was to examine the predictive value of fear extinction (FE) learning ability for inter-individual differences in pain-related behavioral responses, particularly emotional-affective pain aspects, with regard to sex. We subjected adult male and female rats to cued fear learning and FE tests and correlated inter-individual differences with pain responses in models of acute arthritis pain and chronic neuropathic pain. We also investigated sex differences in FE phenotypes for measures of sensory (mechanical withdrawal thresholds) and emotional-affective (open field tests for anxiety-like behaviors and audible and ultrasonic components of vocalizations) pain-related behaviors.

## 2. Materials and Methods

### 2.1. Animals

A total of 215 male and 190 female Sprague-Dawley rats (150–350 g, 6–12 weeks of age) were group-housed in a temperature-controlled room under a 12 h light/dark cycle with unrestricted access to food and water. On each experimental day, rats were transferred from the animal facility and allowed to acclimate to the laboratory for at least 1 h. Experimental procedures were approved by the Institutional Animal Care and Use Committee (IACUC; protocol #14006) at Texas Tech University Health Sciences Center and conformed to the guidelines of the International Association for the Study of Pain (IASP) and of the National Institutes of Health (NIH).

### 2.2. Experimental Protocol

**Figure 1:**
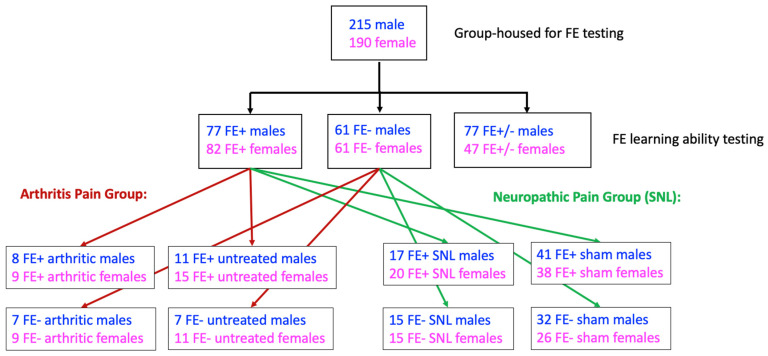
Experimental design. Rats underwent fear conditioning and extinction learning protocols before being separated into FE+ and FE− groups for either the acute arthritis pain (vs. untreated control) groups or the chronic neuropathic pain (vs. sham control) groups. FE: fear extinction.

Naïve rats were subjected to fear conditioning and FE trials. Rats were then randomly assigned to the arthritis pain model (see Section 2.3) or the neuropathic pain model (see Section 2.4). One week later, the neuropathic pain model was induced or sham surgery was performed. Four weeks after surgery, neuropathic pain-related behavioral changes reach a stable plateau in this model [29]. The arthritis pain model was induced in a separate group of rats at the four-week time point to achieve age-matched experimental groups. Behavioral studies were performed four weeks after surgery or 6 h after arthritis induction when behavioral and neurobiological changes are known to reach a maximum plateau [21]. The experimenter was blinded with regard to the FE phenotype, the neuropathic versus sham condition, and the arthritis versus untreated control condition. The experimental design is illustrated in Figure 1.

### 2.3. Arthritis Pain Model

The well-established mono-arthritis pain model mimics the acute phase of the human osteoarthritis condition and was induced in the left knee joint as described in detail previously [21]. Rats were briefly anesthetized with isoflurane (2–3%; precision vaporizer, Harvard Apparatus, Holliston, MA) and a kaolin suspension (4% in sterile saline, 100 μL) was slowly injected into the joint cavity followed by repetitive flexions and extensions of the leg for 15 min. A carrageenan solution (2% in sterile saline, 100 μL) was then injected into the knee joint cavity and the leg was flexed and extended for another 5 min. This treatment paradigm (the K/C arthritis model) reliably produces a localized inflammation in only one knee joint with damage to the cartilage within 1–3 h. K/C arthritis persists for at least a week and is associated with pain behaviors and neural activity changes in the central and peripheral nervous system. Naïve rats that underwent similar handling but did not receive intraarticular injections were used as a control group, as data from our previous studies demonstrated no differences in the behavior of untreated rats and of those that received intraarticular saline injection [34] or needle insertion [35]. This justified the use of naïve rats as an appropriate control for the K/C pain model, which is well established in our laboratories [36,37,38].

### 2.4. Neuropathic Pain Model

The well-established spinal nerve ligation (SNL) model of neuropathic pain [39] was used, which creates stable and long-lasting neuropathic pain behaviors. Rats were anesthetized with isoflurane (2–3%; precision vaporizer, Harvard Apparatus) and underwent sterile surgery where the left L5 spinal nerve was exposed and tightly ligated using 6–0 sterile silk. In the sham-operated control group, the nerve was exposed but not ligated.

### 2.5. Behaviors

#### 2.5.1. Fear Conditioning and Extinction

Fear conditioning and extinction learning tests were conducted using two chambers of a near infrared Video Fear Conditioning System (Med Associates Inc., Fairfax, VT, USA) as described previously [40,41,42]. The conditioning chambers were located inside a sound-attenuating isolation cabinet with a metal grid flooring that was connected to a grid stimulator to administer aversive foot shocks. Two distinct chambers with separate visual, olfactory, tactile, dimensional, and lighting environments were used (context A: white light, no fan in chamber, metal grid on chamber floor, lights on in experimental room, rat transported to chamber in transparent box, chamber cleaned with 50% ethanol; context B: near-infrared (NIR) light, fan on in chamber, flat chamber floor, lights off in experimental room, rat transported to chamber in opaque box, chamber cleaned with 70% isopropanol, colored insert with 3 drops of peppermint oil added to alter olfactory environment and physical dimensions). Day 1 consisted of the training phase where rats were habituated to the training chamber (context A) and allowed to explore freely for 5 min, followed by fear conditioning that consisted of a foot shock (0.7 mA, 2 s; the unconditioned stimulus, USA) delivered during the final 2 s of an auditory stimulus (white noise, 80 dB, 4.5 kHz, 30 s; the conditioned stimulus, CS). Two CS-US pairings were used (intertone interval, ITI, 120 s). On day 2, rats were placed in a different chamber (context B) and were habituated for 5 min, followed by extinction training (30 CSs, ITI 5 s). A mounted video camera in the conditioning chambers was used to record the behavior of each rat. Freezing behavior (expressed as a percentage of each 30 s period) was analyzed and quantified using Video Freeze software (Med Associates Inc.) as the conditioned response. Based on their FE learning ability, rats were classified into strong (FE+), “normal” (FE+/−), and weak (FE−) FE groups as determined by evidence of diminishing (below 50%) freezing responses during Phase I (before 600 s), Phase II (600–900 s), or Phase III (after 900 s) of extinction training (see the “Results” section for details). Rats in the two extreme groups (FE+ and FE−) were selected for further behavioral testing and randomly assigned to groups in the arthritis pain model (untreated FE+, untreated FE−, arthritis FE+, and arthritis FE−) or in the neuropathic pain model (sham FE+, sham FE−, SNL FE+, and SNL FE−). Four weeks after SNL or sham surgery, or 6 h after arthritis induction in an age-matched model, behavioral assays (see next paragraphs) were performed.

#### 2.5.2. Mechanosensitivity

Rats were briefly anesthetized with isoflurane (2–3%; precision vaporizer, Harvard Apparatus) and were placed slightly restrained in a customized recording chamber that permitted access to the hindlimbs (U.S. Patent 7,213,538) for stable testing. Hindlimb withdrawal thresholds were evaluated after recovery from anesthesia and after habituation to the recording chamber for 30 min. Hindlimb withdrawal thresholds were evaluated using calibrated forceps with a force transducer whose output was displayed in grams on an LED screen. The calibrated forceps were used to gradually compress the left knee joint (arthritis pain model) or the left hindpaw (neuropathic pain model) with a continuously increasing intensity until a withdrawal reflex was evoked as described in our previous studies [21,35,37,38,43,44,45]. The withdrawal threshold, defined as the force required to evoke a reflex response, was calculated using the average value from 2 to 3 trials.

#### 2.5.3. Emotional Responses

Components of vocalizations in the audible (20 Hz–16 kHz) and ultrasonic (25 ± 4 kHz) ranges were simultaneously measured after hindlimb withdrawal assays using an automatic computerized vocalization system consisting of a full-spectrum USB ultrasound microphone (max sampling rate: 384 kHz) and UltraVox XT four-channel recording and analysis system (Noldus Information Technology, Leesburg, VA, USA). Rats were briefly anesthetized with isoflurane (2–3%; precision vaporizer, Harvard Apparatus) and placed in the customized recording chamber for stable recordings of vocalizations evoked by natural stimulation. After the rat recovered from anesthesia and habituated to the recording chamber for 30 min, hindlimb withdrawal thresholds were evaluated (see Section 2.5.2) and the calibrated forceps with a force transducer were used for vocalization assays. Vocalizations were evoked by a brief (10 s), continuous noxious stimulus applied to the left knee joint (arthritis pain model; stimulus: 1500 g/30 mm^2^) or to the left hindpaw (neuropathic pain model; stimulus: 500 g/6 mm^2^) as described in our previous studies [20,28,29,37,38,42]. Vocalizations were automatically detected for 1 min and total durations of audible and ultrasonic components of vocalizations following the onset of mechanical stimulus were analyzed using UltraVox 3.2 software (Noldus Information Technology). For vocalization analyses, audible calls were labeled using frequency ranges of 20 Hz–16 kHz and ultrasonic components of calls were labeled using frequency ranges of 21–29 kHz. The following call descriptions were also specified: minimum amplitude, 50 units; minimum duration, 1 ms; maximum duration, 2000 ms; minimum gap between calls, 1 ms. Calls that fit these criteria were detected for each recording. At the conclusion of each experiment, call statistics for each recording were exported as a text file. The duration (in ms) for each individual call was summed for each 1 min recording period to give the total duration of audible and ultrasonic components of vocalizations for each rat.

#### 2.5.4. Anxiety-Like Behavior

Animal movements within the open field test (OFT) were used to measure anxiety-like behavior. Exploratory behavior in the central or peripheral zones of an arena (70 cm × 70 cm) with acrylic walls (height, 45 cm) was recorded for 15 min using a computerized video tracking and analysis system (EthoVision XT 11 software, Noldus Information Technology) as described previously [42,46]. Time spent in the center of the arena (35 cm × 35 cm) was calculated during the first 5 min. Avoidance of the center of the arena is interpreted to suggest anxiety-like behavior [42,46,47,48].

### 2.6. Statistical Analysis

All averaged values are presented as the mean ± SE. Statistical significance was accepted at the level *p* < 0.05. GraphPad Prism 9.0 software was used for all statistical analyses. Statistical analyses were performed on the raw data. For multiple comparisons, a two-way analysis of variance (ANOVA) was used with Bonferroni post hoc tests. 

## 3. Results

### 3.1. Inter-Individual and Sex Differences in FE Learning Ability of Naïve Male and Female Rats

Fear learning and FE are well-established models of aversive learning that have been used to correlate behavior with neural structure and function, which involve cortico-limbic circuits centered on the amygdala [15]. We previously reported that the identification of distinct behavioral phenotypes based on FE ability in naïve male rats can serve as a predictor for inter-individual differences in pain sensitivity and amygdala neuronal activity in chronic neuropathic pain [42]. Here, we chose to examine whether a similar correlation existed between FE learning ability and acute arthritis pain-related behaviors and if this predictive value could be expanded to include both sexes.

**Figure 2:**
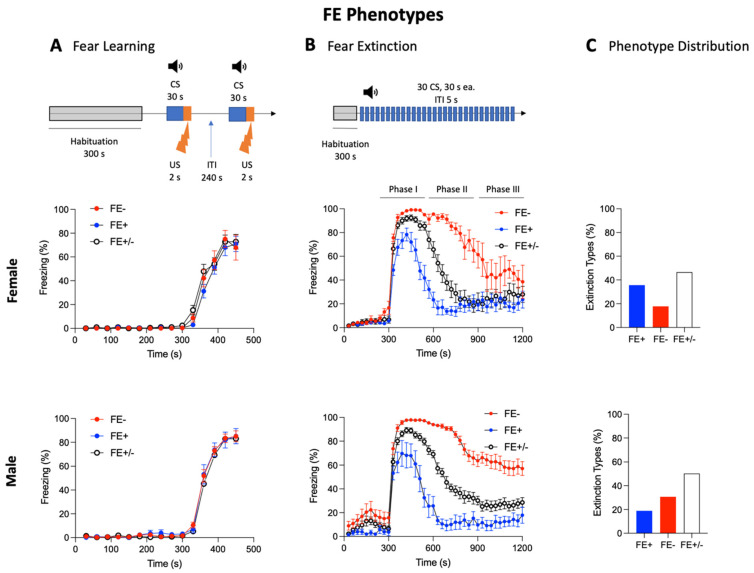
Inter-individual and sex differences in fear extinction learning ability in naïve female and male rats. Fear conditioning on Day 1 (**A**) and extinction (**B**) tests were conducted using two distinct context chambers. (**A**) Fear conditioning on Day 1—rats were habituated to context A followed by fear conditioning (2 CS-US pairs, see Section 2.5.1). The diagram illustrates the experimental protocol. Symbols in the line graph show freezing responses expressed in percent per 30 s segment during fear conditioning with 2 CS-US pairings. (**B**) Fear extinction learning on Day 2—rats were habituated to context B followed by extinction training (30 CSs, no US). The diagram illustrates the experimental protocol. Symbols in the line graph show freezing responses to tone (CS) expressed in percent per 30 s segment. (**C**) Bar histograms show the distribution of rats with strong (FE+), “normal” (FE+/−), and weak (FE−) fear extinction. The population (%) of FE+ was larger in female rats compared to male rats. For details, see the “Methods” and “Results” sections. CS: conditioned stimulus; US: unconditioned stimulus; ITI: intertone interval; FE: fear extinction.

Fear learning and FE were measured in 215 male and 190 female naïve rats (see Section 2.1 and Section 2.5.1). During the fear learning session on day 1 of fear conditioning, rats showed minimal freezing behavior during the habituation phase under context A, indicating normal locomotor activity. All rats developed freezing responses after two pairings of CS (white noise, 80 dB, 4.5 kHz, 30 s) and US (0.7 mA foot shock, 2 s) (Figure 2A). During the fear training session on day 2, three groups emerged in both sexes based on differences in the time course and magnitude of declining freezing behavior in the absence of a foot shock (the US) (Figure 2B). For females, 36 rats (35.6%) exhibited a rapid (before 600 s; Phase I) decline in freezing to levels below 50% (per 30 s CS segment), reflecting strong FE learning ability (FE+), while 18 rats (17.8%) maintained freezing levels above 50% past 900 s (Phase III), indicating weak FE learning ability (FE−). The remaining 47 rats (46.5%) showed a decline to below 50% freezing levels between 600 and 900 s (Phase II) of the FE session and were classified as exhibiting “normal” FE learning ability (FE+/−). Males exhibited a different distribution of phenotypes, where 29 rats (19.8%) showed strong FE learning ability (FE+), 47 rats (30.7%) showed weak FE learning ability (FE−), and the remaining 77 rats (50.3%) showed normal FE learning ability (FE+/−) (Figure 2C). Female FE− rats showed a significantly higher percent freezing per 30 s CS segment than those in the female FE+ group (*p* < 0.0001, F_1,2080_ = 512.8, two-way ANOVA; Bonferroni post hoc test results are shown in Figure 2B). Similarly, males in the FE− group showed a significantly higher percent freezing per 30 s CS segment than males in the FE+ group (*p* < 0.001, F_1,2960_ = 1372, two-way ANOVA; Bonferroni post hoc test results are shown in Figure 2B). Interestingly, FE+ males exhibited significantly lower percent freezing per 30 s CS segment than FE+ females (*p* < 0.01, F_12,520_ = 12.42, two-way ANOVA with Bonferroni post hoc tests) while FE− males showed significantly higher percent freezing per 30 s CS segment than FE− females (*p* < 0.0001, F_12,520_ = 22.75, two-way repeated-measures ANOVA with Bonferroni post hoc tests). Importantly, no differences in percent freezing were observed between the three groups for either sex during the habituation phases of the fear learning (Figure 2A) or the fear extinction (Figure 2B) sessions.

### 3.2. Inter-Individual and Sex Differences in Arthritis Pain-Related Behaviors of FE+ and FE− Rats

**Figure 3:**
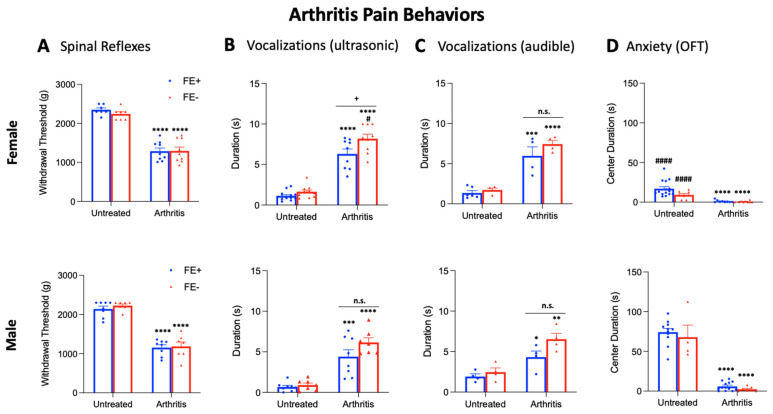
Inter-individual and sex differences in arthritis pain-related behaviors of FE+ and FE− rats. (**A**) Mechanical thresholds tested in untreated control rats and arthritic rats (6 h post-induction) showed no significant differences between FE− (female, *n* = 7; male, *n* = 7) and FE+ (female, *n* = 7; male, *n* = 8) untreated rats or between FE− (female, *n* = 9; male, *n* = 7) and FE+ (female, *n* = 9; male, *n* = 8) arthritic rats, but arthritic FE− and FE+ rats had significantly lower withdrawal thresholds than their untreated controls. **** *p* < 0.0001, ANOVA with Bonferroni post hoc tests (see the “Results” section). (**B**,**C**) Duration (s) of ultrasonic and audible vocalizations, respectively, evoked by a brief (10 s) noxious (1500 g/30 mm^2^) mechanical compression of the knee. Significant differences in ultrasonic (but not audible) vocalizations were found between FE− (*n* = 9) and FE+ (*n* = 9) female arthritic rats but not between FE− (*n* = 7) and FE+ (*n* = 8) male arthritic rats or between untreated FE− (female, *n* = 11; male, *n* = 7) and FE+ (female, *n* = 13; male, *n* = 8) rats. For both sexes, arthritic rats had significantly increased vocalizations compared to their untreated controls. n.s.: non-significant; ^+^ *p* < 0.05; ^#^ *p* < 0.05; * *p* < 0.05; ** *p* < 0.01; *** *p* < 0.001; **** *p* < 0.0001, ANOVA with Bonferroni post hoc tests (see the “Results” section). (**D**) Center duration (s) in the OFT was significantly lower in arthritic FE− (female, *n* = 9; male, *n* = 7) and FE+ (female, *n* = 9; male, *n* = 13) rats compared to the untreated FE− (female, *n* = 8; male, *n* = 7) and FE+ (female, *n* = 15; male, *n* = 11) control rats. No differences were found between FE− and FE+ rats in the untreated control or arthritic groups for either sex. ^####^ *p* < 0.0001; **** *p* < 0.0001, ANOVA with Bonferroni post hoc tests (see the “Results” section). Bar histograms show means ± SEM. FE: fear extinction; OFT: open field test. Asterisk (*) indicates comparison to untreated group; plus sign (+) indicates comparison between phenotypes; pound sign (#) indicates comparison between sexes.

Next, we examined whether inter-individual and sex differences in FE learning ability would correspond with behavioral differences for males and females in an arthritis pain model (K/C arthritis, see Section 2.3) and/or in the untreated control condition. Male and female rats from the FE+ and FE− groups were selected for further behavioral testing and randomly assigned to either the K/C arthritis group or the untreated control group. Five weeks later (corresponding with an age-matched neuropathic pain group), arthritis was induced, and 6 h later, the following behavioral assays were performed: nocifensive reflexes (mechanosensitivity, Figure 3A) and ultrasonic and audible components of vocalizations (emotional responses, Figure 3B,C) evoked by mechanical compression of the knee joint, and the OFT (anxiety-like behavior, Figure 3D). 

No significant differences in mechanical withdrawal thresholds were found between untreated FE+ rats (female, *n* = 7; male, *n* = 8) or untreated FE− rats (female, *n* = 7; male, *n* = 7) for either sex (Figure 3A). Similarly, no significant differences in mechanosensitivity were found between FE+ rats (female, *n* = 9; male, *n* = 8) or FE− rats (female, *n* = 9; male, *n* = 7) in the arthritis pain model for either sex. However, mechanical withdrawal thresholds were significantly lower for arthritic female FE+ and FE− rats and for arthritic male FE+ and FE− rats compared to their untreated controls (*p* < 0.0001, as shown in Figure 3A), suggesting that both types of rats developed hypersensitivity in the pain model. No significant differences in mechanical withdrawal thresholds were found between female FE+ rats and male FE+ rats or between female FE− rats and male FE− rats for either the arthritis or untreated control groups. For the statistical analyses of mechanical withdrawal thresholds in the four female experimental groups and the four male experimental groups, ANOVA with Bonferroni post hoc tests was used (female, F_3,28_ = 53.09; male, F_3,26_ = 57.02). 

For the ultrasonic and audible components of vocalizations (Figure 3B,C), no significant differences were found between untreated FE+ rats (female, *n* = 13; male, *n* = 8) and untreated FE− rats (female, *n* = 11; male, *n* = 7) for either sex. However, the total duration of vocalizations was significantly higher in female FE− rats (*n* = 9) than female FE+ rats (*n* = 9) in the arthritis pain model (*p* < 0.05, Figure 3B). No significant differences were found in the durations of audible components of vocalizations of these groups or in ultrasonic and audible components of vocalizations of male FE+ rats (*n* = 8) and male FE− rats (*n* = 7) in the arthritis group, though there was a non-significant trend (ultrasonic, *p* = 0.1988; audible, *p* = 0.1398). Total durations of ultrasonic and audible components of vocalizations were significantly increased for arthritic female FE+ and FE− rats and for arthritic male FE+ and FE− rats compared to their untreated controls (*p* < 0.05–0.0001, as shown in Figure 3B,C). Female FE− rats had significantly increased durations of ultrasonic but not audible components of vocalizations compared to male FE− rats (*p* < 0.05, as shown in Figure 3B) in the arthritis model. No differences were seen for durations of ultrasonic and audible components of vocalizations between female FE+ and male FE+ groups (untreated control or arthritis). Together, the data suggest that all groups developed emotional responses to arthritis pain, though it emerged most prominently for female FE− rats. For the statistical analyses of vocalization durations in the four female experimental groups and the four male experimental groups, ANOVA with Bonferroni post hoc tests was used (ultrasonic: female, F_3,38_ = 80.32, and male, F_3,26_ = 23.49; audible: female, F_3,14_ = 27.75, and male, F_3,12_ = 11.88). 

In the OFT (Figure 3D), no significant difference in arena center duration was found between untreated FE+ rats (female, *n* = 15; male, *n* = 11) and untreated FE− rats (female, *n* = 8; male, *n* = 7) for either sex. Similarly, no significant differences in center duration were found between FE+ rats (female, *n* = 9; male, *n* = 13) and FE− rats (female, *n* = 9; male, *n* = 7) in the arthritis pain model for males or females. In the arthritis pain groups, female FE+ and FE− rats and male FE+ and FE− rats spent significantly less time in the center of the arena compared to their untreated controls (*p* < 0.0001, Figure 3D), suggesting all groups developed increased anxiety-like behavior. However, in the untreated control group, female FE+ and FE− rats spent significantly less time in the arena center compared to male FE+ and FE− rats, respectively (*p* < 0.0001, as shown in Figure 3D), suggesting higher baseline anxiety levels for females of both phenotypes. No significant differences in center duration were seen between females and males in the arthritis pain model for either phenotype. Importantly, no significant differences in locomotor activity were observed between the arthritis pain group and the untreated control group (*p* = 0.7327, Figure 3D), indicating that differences in anxiety-like behavior were not due to a reduction in spontaneous activity following arthritis induction. For the statistical analyses of OFT center duration in the four female experimental groups and the four male experimental groups, ANOVA with Bonferroni post hoc tests was used (female, F_3,37_ = 16.94; male, F_3,31_ = 72.79).

### 3.3. Inter-Individual and Sex Differences in Neuropathic Pain-Related Behaviors of FE+ and FE− Rats

**Figure 4:**
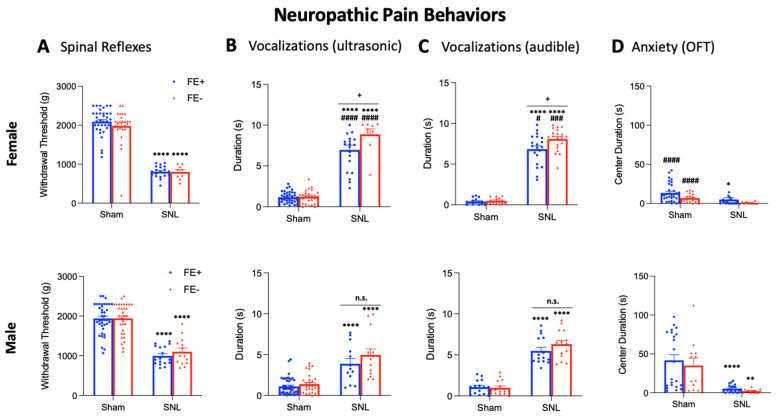
Inter-individual and sex differences in neuropathic pain-related behaviors of FE+ and FE− rats. (**A**) Mechanical thresholds tested in sham and chronic neuropathic SNL rats (4 weeks post-induction) showed no significant differences between FE− (female, *n* = 26; male, *n* = 32) and FE+ (female, *n* = 35; male, *n* = 40) sham rats or between FE− (female, *n* = 9; male, *n* = 14) and FE+ (female, *n* = 20; male, *n* = 17) SNL rats, but SNL FE− and FE+ rats had significantly lower withdrawal thresholds than their sham controls. **** *p* < 0.0001, ANOVA with Bonferroni post hoc tests (see the “Results” section). (**B**,**C**) Duration (s) of of ultrasonic and audible vocalizations, respectively, evoked by a brief (10 s) noxious (1500 g/6 mm^2^) mechanical compression of the affected hindpaw. Significant differences in ultrasonic and audible vocalizations were found between female FE− (*n* = 15) and FE+ (*n* = 20) SNL rats but not between male FE− (*n* = 15) and FE+ (*n* = 15) SNL rats or between FE− (female, *n* = 26; male, *n* = 32) and FE+ (female, *n* = 38; male, *n* = 41) sham rats. For both sexes, SNL rats had significantly increased vocalizations compared to their sham controls. n.s.: non-significant; ^+^ *p* < 0.05; ^#^ *p* < 0.05; ^###^ *p* < 0.001; ^####^ *p* < 0.0001; **** *p* < 0.0001, ANOVA with Bonferroni post hoc tests (see the “Results” section). (**D**) Center duration (s) in the OFT was significantly lower in FE− (female, *n* = 9; male, *n* = 13) and FE+ (female, *n* = 20; male, *n* = 20) SNL rats compared to their FE− (female, *n* = 19; male, *n* = 12) and FE+ (female, *n* = 33; male, *n* = 22) sham controls. No differences were found between FE− and FE+ rats in the sham or SNL groups for either sex. ^####^ *p* < 0.0001; * *p* < 0.05; ** *p* < 0.01; **** *p* < 0.0001, ANOVA with Bonferroni post hoc tests (see the “Results” section). Bar histograms show means ± SEM. FE: fear extinction; OFT: open field test; SNL: spinal nerve ligation. Asterisk (*) indicates comparison to untreated group; plus sign (+) indicates comparison between phenotypes; pound sign (#) indicates comparison between sexes.

As we previously reported that FE learning ability may serve as a predictor for neuropathic pain-related behaviors in male rats [42], we next sought to determine whether inter-individual differences in FE learning ability may also translate into behavioral differences for females in a neuropathic pain model (SNL, see Section 2.4) and/or in the sham control condition. Male and female FE+ and FE− rats were randomly assigned to the neuropathic pain group or sham group, and four weeks after SNL or sham surgery, the same behavioral assays were performed in these animals: nocifensive reflexes (Figure 4A) and ultrasonic and audible components of vocalizations (Figure 4B,C) evoked by mechanical compression of the hindpaw, and the OFT (Figure 4D).

Mechanical withdrawal thresholds showed no significant differences between sham FE+ rats (female, *n* = 35; male, *n* = 40) and sham FE− rats (female, *n* = 26; male, *n* = 32) for either sex (Figure 4A). Similarly, in the neuropathic pain model, there were no significant differences found in withdrawal thresholds between FE+ rats (female, *n* = 20; male, *n* = 17) and FE− rats (female, *n* = 9; male, *n* = 14) for either sex. Both female and male FE+ and FE− rats in the neuropathic pain model showed significantly lower mechanical thresholds compared to their sham controls (*p* < 0.0001, as shown in Figure 4A), suggesting both types of rats developed neuropathic hypersensitivity. No significant differences in mechanical withdrawal thresholds were found between female FE+ and male FE+ rats or between female FE− and male FE− rats for either the neuropathic pain or sham control groups. For the statistical analyses of mechanical withdrawal thresholds between the four female experimental groups and the four male experimental groups, ANOVA with Bonferroni post hoc tests was used (female, F_3,86_ = 92.25; male, F_3,99_ = 46.40). 

**Figure 5:**
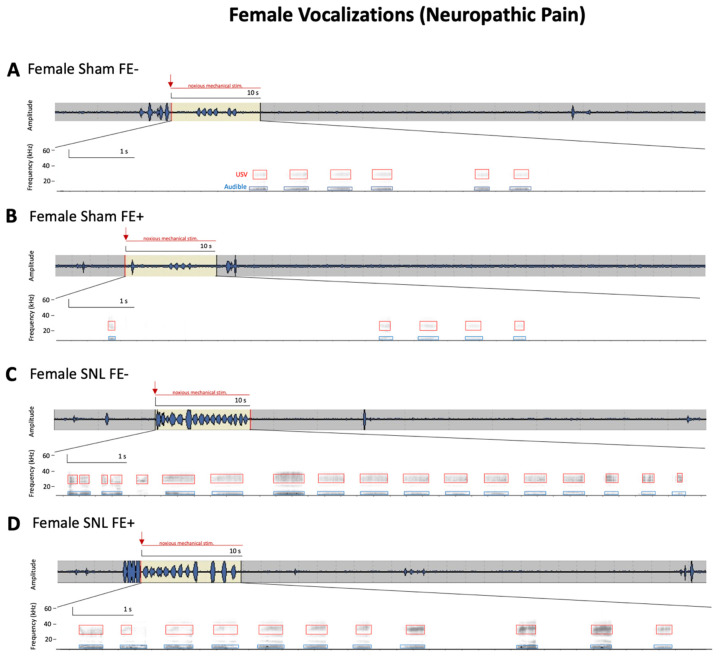
Representative audible and ultrasonic vocalizations from phenotyped female rats in the SNL model of neuropathic pain. Original real-time waveform and spectrogram recordings of vocalizations evoked in response to brief (10 s) noxious (1500 g/6 mm^2^) mechanical stimulation of the affected hindpaw 4 weeks after induction of sham (**A**,**B**) or SNL (**C**,**D**) surgery in phenotyped female rats. For details, see Section 2.5.3. Mechanical stimuli were applied to the hindpaw in each recording period, as indicated by the highlighted yellow section of the waveform (upper panel, red arrow indicates initiation of noxious stimulus application); the total duration of the recording is 1 min. Boxes (events) in the spectrogram (lower panel) represent the presence of audible (blue; 20 Hz–16 kHz) and ultrasonic (red; 25 ± 4 kHz) vocalizations during the 10 s application of mechanical stimuli. Female FE− sham rats (**A**) showed more vocalization events in response to noxious stimulus than female FE+ sham rats (**B**). Female FE− SNL rats (**C**) showed more vocalization events in response to noxious stimulus than female FE+ SNL rats (**D**). FE: fear extinction; SNL: spinal nerve ligation; USV: ultrasonic vocalizations.

**Figure 6:**
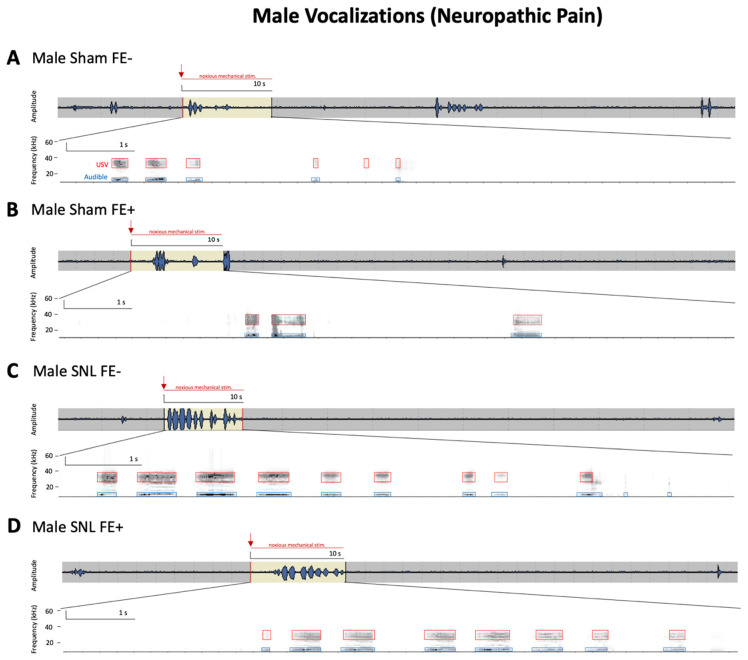
Representative audible and ultrasonic vocalizations from phenotyped male rats in the SNL model of neuropathic pain. Original real-time waveform and spectrogram recordings of vocalizations evoked in response to brief (10 s) noxious (1500 g/6 mm^2^) mechanical stimulation of the affected hindpaw 4 weeks after induction of sham (**A**,**B**) or SNL (**C**,**D**) surgery in phenotyped male rats. For details, see Section 2.5.3. Mechanical stimuli were applied to the hindpaw in each recording period, as indicated by the highlighted yellow section of the waveform (upper panel, red arrow indicates initiation of noxious stimulus application); the total duration of the recording is 1 min. Boxes (events) in the spectrogram (lower panel) represent the presence of audible (blue; 20 Hz–16 kHz) and ultrasonic (red; 25 ± 4 kHz) vocalizations during the 10 s application of mechanical stimuli. Male FE− sham rats (**A**) showed more vocalization events in response to noxious stimulus than male FE+ sham rats (**B**). Male FE− SNL rats (**C**) showed more vocalization events in response to noxious stimulus than male FE+ SNL rats (**D**). FE: fear extinction; SNL: spinal nerve ligation; USV: ultrasonic vocalizations.

For ultrasonic and audible components of vocalizations (Figure 4B,C), no significant differences in duration were found between sham FE+ rats (female, *n* = 38; male, *n* = 41) and sham FE− rats (female, *n* = 26; male, *n* = 32) for either sex. However, the total durations of ultrasonic and audible components of vocalizations were significantly increased in female FE− rats (*n* = 15) compared to female FE+ rats (*n* = 20) in the neuropathic pain model (*p* < 0.05, as shown in Figure 4B,C). No significant differences in the durations of audible or ultrasonic components of vocalizations were found for male FE+ rats (*n* = 15) and male FE− rats (*n* = 15) in neuropathic pain. Both female and male FE+ and FE− rats in the neuropathic model had significantly increased durations of ultrasonic and audible components of vocalization compared to their sham controls (*p* < 0.0001, as shown in Figure 4B,C). In the neuropathic pain group, FE+ female rats had significantly greater durations of ultrasonic and audible components of vocalizations than FE+ male rats (ultrasonic: *p* < 0.0001, as shown in Figure 4B; audible: *p* < 0.05, as shown in Figure 4C) and FE− female rats had significantly greater durations of ultrasonic and audible components of vocalizations than FE− male rats (ultrasonic: *p* < 0.0001, as shown in Figure 4B; audible: *p* < 0.001, as shown in Figure 4C). Together, the data suggest that while all groups developed emotional responses to neuropathic pain, this occurred most prominently for FE− females. Individual examples of real-time waveforms and spectrogram recordings for phenotyped female and male SNL and sham control rats are shown in Figure 5 and Figure 6. Though not reported in this study, our recordings (see Figure 5 and Figure 6) suggest that similar differences between sexes and phenotypes may be observed with regard to the total number of vocalizations. Both the total duration [20,24] and the total number of calls [27,49] have been utilized as effective measures of behavioral responses in the context of pain. For the statistical analyses of ultrasonic and audible components of vocalizations between the four female experimental groups and the four male experimental groups, ANOVA with Bonferroni post hoc tests was used (ultrasonic: female, F_3,89_ = 125.9, and male, F_3,96_ = 25.90; audible: female, F_3,66_ = 177.9, and male, F_3,56_ = 66.50). 

In the OFT (Figure 4D), no significant differences in arena center duration were found between sham FE+ rats (female, *n* = 33; male, *n* = 22) and sham FE− rats (female, *n* = 19; male, *n* = 12). Similarly, no differences in arena center duration were found between FE+ rats (female, *n* = 20; male, *n* = 20) and FE− rats (female, *n* = 9; male, *n* = 13) in the neuropathic pain model. Female FE+ (but not FE−) rats and male FE+ and FE− rats in the neuropathic pain group spent significantly less time in the center of the arena compared to their sham controls (*p* < 0.05–0.0001, as shown in Figure 4D). In the sham control group, female FE+ and FE− rats spent significantly less time in the center of the arena compared to male FE+ and FE− rats (*p* < 0.0001, as shown in Figure 4D), suggesting higher anxiety levels for females at baseline, as also seen in naïve rats (see Figure 3D). Importantly, there were no significant differences in locomotor activity between the neuropathic pain and sham control groups (*p* = 0.8120, Figure 4D) or between the sham control group and the untreated control group for the arthritis model (*p* = 0.4292, see Figure 3D), indicating that the observed differences in anxiety-like behavior were not due to a reduction in spontaneous activity following surgical procedures. For the statistical analyses of OFT center duration between the four female experimental groups and the four male experimental groups, ANOVA with Bonferroni post hoc tests was used (female, F_3,78_ = 6.119; male, F_3,63_ = 11.53).

## 4. Discussion

This study explored the predictive value of FE learning ability in sensory and affective pain-related behaviors for male and female animals in an acute arthritis and a chronic neuropathic pain model. We previously showed a positive correlation between FE learning ability and neuropathic pain behaviors in adult male rats [42], but it is unclear if these are also found in acute pain conditions and whether female rats exhibit a similar association. The key novelties of this study are the identification of distinct behavioral phenotypes based on FE learning ability for both sexes, with vocalizations being the most effective indicators, and that these behavioral phenotypes show striking differences between male and female rats in both pain models. 

Fear learning and extinction assays were selected as approaches to identifying inter-individual differences in pain-related behaviors because these are well-established models for correlating animal behavior with neural structure and function [15]. Previous studies from our lab and others have reported the separation of fast and slow recovery phenotypes based on freezing levels during FE that correlate with differences in anxiety-like behavior [16,42,50,51,52]. At the clinical level, inter-individual differences in fear response modulation and generalization have been linked to increased vulnerability in the development of anxiety disorders and post-traumatic stress disorder (PTSD) [15,17,53,54,55], and patients with anxiety disorders, PTSD, and obsessive-compulsive disorder (OCD) have exhibited delayed and/or reduced FE or extinction recall [14,56,57,58,59]. A previous epidemiological study reported that most individuals who experience trauma recover, with only a subset going on to develop a psychopathology such as depression or anxiety [60]. Similarly, chronic widespread pain develops in only 10% of the population [61]. However, a major goal of preclinical research is to provide insights into neural processes and behaviors that can predict susceptibility versus resistance to a disorder. This requires the study of neural variability patterns that differ from the central tendency. Thus, we chose to focus on (representatives of) the groups at the two ends of the spectrum (weak FE learning ability (FE−), considered to be “susceptible” rats, versus strong FE learning ability (FE+), considered to be “resistant” rats) within this study instead of including the larger, “normal” FE+/− group.

Little has been studied with regard to sex differences in classic fear conditioning and extinction models. This is an important knowledge gap, as females have twice the lifetime rates of depression and anxiety disorders [62], and human imaging studies revealed structural and functional sex differences in anxiety-relevant brain regions [63]. One preclinical study found that, while fast and slow extinction phenotypes could be identified for both sexes, there were no observable differences between males and females in freezing levels during fear conditioning or extinction [64]. Others have reported impairments in FE recall for female rats when compared to male rats [65,66]. However, several studies have reported that females have greater FE rates when compared to males [67,68,69,70]. A recent review suggested that sex hormones may play an important role in conditioned FE, as estrous cycle influences may affect female FE mechanisms [71]. The results of this study are consistent with those from the literature that showed gonadectomized males spent a greater amount of time freezing than gonadectomized females, a pattern that was not affected by estradiol administration [68]. Another study suggested that endogenous estrogen did not affect FE behavior in female rats or naturally cycling women [72]. Therefore, it is unlikely that the estrous cycle significantly affected the freezing levels of females in our study. 

Inter-individual differences have been well-documented for pain and pain modulation [5]. Neurobiological mechanisms, including emotional network plasticity, may link pain and fear [11]. This relationship has been explored with regard to the corticolimbic system [73,74], and in particular, the amygdala, a limbic structure that has emerged as a key player in both fear and anxiety networks [75,76,77,78] and in the emotional-affective dimensions of pain and pain modulation [8,10,79]. Corticolimbic characteristics involving the amygdala determine the risk of chronic pain and mediate the effects of depression and negative affect on chronic pain [80]. Human studies have investigated the role of the amygdala in pain and fear interactions [73,81], and the amygdala has been implicated in fear-conditioned analgesia in a preclinical setting [82,83,84]. The amygdala has been implicated in pain-like behaviors [85,86,87], anxiety-like behaviors [88,89,90], and in fear learning [12,91]. Pain-related neuroplastic changes lead to hyperexcitability in amygdala output neurons [10], driving pain behaviors in both acute [92,93] and chronic [29,94] pain models. Sex differences with regard to pain conditions have long been recognized, with females greatly outnumbering males as chronic pain patients [6]. However, sex differences in pain-related amygdala neuroplasticity are largely unknown, though one clinical study reported sex differences in resting-state amygdala subnuclei connectivity patterns as a potential explanation for the increased prevalence of conditions of negative affect in women [95]. Even less has been explored about inter-individual and sex differences in fear learning and FE with regard to pain and pain modulation, though a clinical study reported sex differences in pain-related fear conditioning [96]. Ultrasonic vocalizations were previously associated with increased neuronal activity in brain regions regulating fear and anxiety, including the amygdala [97], and have been demonstrated to be an effective indicator of emotional status in pain models [20,21,27]. As ultrasonic vocalizations demonstrated the most striking inter-individual and sex differences in pain-related behaviors for both an acute and a chronic model, insight into potential sexual dimorphisms of pain-related amygdala neuroplasticity is warranted.

The intricate relationship between pain modulation and fear neurocircuitry and mechanisms, particularly in relation to potential discrepancies regarding sex differences, led us to test the hypothesis that FE learning ability can predict pain-related behaviors in both acute (arthritis) and chronic neuropathic (SNL) models of pain, and that these behaviors may differ between males and females. In the present study, distinct behavioral phenotypes differed according to sex in their FE but not fear learning ability. There were no differences at baseline between mechanosensitivity (spinal reflex thresholds) and emotional-affective responses (vocalizations), but females exhibited increased baseline anxiety-like behavior (OFT) compared to males in both the untreated and sham-treated control groups (see Figure 3D and Figure 4D). This confirms findings from the literature that males spent the same or increased time in the center of the OFT compared to females at baseline [3,98], though one study found no sex difference in OFT anxiety-like behavior in a chronic spinal nerve transection pain model [99]. FE+ and FE− phenotypes showed differences in the magnitude of emotional-effective responses not only in the neuropathic pain model, as we previously reported [42], but also in the arthritis pain model (see Figure 3B,C and Figure 4B,C). Additionally, females exhibited significantly increased audible and ultrasonic components of vocalizations compared to males in both of the tested pain models. To the best of our knowledge, sex differences in pain-related vocalizations in the context of FE learning have not been reported. One preclinical study found that male rats vocalized more than female rats despite females exhibiting lower freezing levels during FE, although this effect was strain-specific and did not include any pain models [100]. The novelty here is the identification of sex-specific differences in behavioral phenotypes, which corresponds to sexual dimorphisms in pain-related vocalizations regardless of pain model. 

Sonic vocalizations, if emitted with large force and volume, may produce overtones that reach into the ultrasonic frequency range. A note of consideration in the present study is that the ultrasonic components (harmonics) of audible vocalizations presented here cannot be regarded as true ultrasonic aversive vocalizations as rats cannot emit sonic and ultrasonic calls at the same time. However, our results show that harmonic components of vigorous audible vocalizations showed an interesting harmonic spectrum, possibly with additional overtones. Because some of the overtones may depart from the whole multiples of the fundamental frequency, the harmonics and overtones show reinforcement at higher frequencies, creating ultrasonic components of the audible calls that are clearly visible in the spectrograms. Ultrasonic components of vocalizations are of long duration, consistent with the duration of audible calls. Simultaneous audible and ultrasonic vocalization components were demonstrated in response to an acute painful stimulus (tail snip) [101]. Ultrasonic harmonics that were previously reported demonstrated a different duration and lower frequency than presented here [102]. Though the emission of 22 kHz ultrasonic vocalizations has been reported to occur after a significant delay [103,104,105], in this study, both audible and ultrasonic components were evoked by a continuously present mechanical stimulus for 10 s as opposed to the brief electrical stimuli used in other studies. Repeated vocalizations may be triggered by the continuous noxious stimulus, and thus, latency assessment is not possible with this approach. The use of audible vocalizations in both the audible and ultrasonic ranges, particularly in correlation with other behavioral measures, is a useful measure of pain levels and emotional responses to pain.

The current study provides the rationale for the inter-individual- and sex-specific analysis of synaptic and cellular mechanisms within the amygdala. Future research may address neuroplastic differences between males and females in the context of pain and fear learning, potentially providing insight into the increased prevalence of anxiety, PTSD, and pain in female patients and supporting patient-specific therapeutic strategies for these disorders [15].

## 5. Conclusions

The data may suggest sexual dimorphisms in FE learning ability that have a predictive value for pain-related behavioral changes, particularly among emotional-affective pain aspects, in both an acute and a chronic pain model. Rats with weak FE learning ability showed an increased magnitude of both arthritic- and neuropathic-pain related affective rather than sensory behaviors, with females demonstrating greater inter-individual differences in affective pain behaviors than males. Vocalizations are strong indicators of inter-individual and sex differences in pain models, particularly in chronic neuropathic pain, whereas no such differences were found for mechanosensitivity, and anxiety-like behaviors showed only baseline differences. The increased correlation between FE learning ability and affective pain-related behaviors in female compared to male rats may be facilitated by amygdala pain mechanisms, though further investigation into sex-specific synaptic and cellular neurobiological mechanisms is warranted.

## Data Availability

All data generated or analyzed during this study are included in this published article. Data files used for this manuscript are available via a direct and reasonable request to the corresponding author and approval from Texas Tech University Health Sciences Center (TTUHSC).
